# Online Measurement of Microembolic Signal Burden by Transcranial Doppler during Catheter Ablation for Atrial Fibrillation—Results of a Multicenter Trial

**DOI:** 10.3389/fneur.2017.00131

**Published:** 2017-04-05

**Authors:** Christian von Bary, Thomas Deneke, Thomas Arentz, Anja Schade, Heiko Lehrmann, Sabine Fredersdorf, Dobri Baldaranov, Lars Maier, Felix Schlachetzki

**Affiliations:** ^1^Department of Cardiology, Rotkreuzklinikum München, Munich, Germany; ^2^Department of Internal Medicine II, University Hospital Regensburg, Regensburg, Germany; ^3^Department of Electrophysiology, Heart Center Bad Neustadt, Bad Neustadt, Germany; ^4^Department of Cardiology and Angiology, Heart Center Bad Krozingen, Bad Krozingen, Germany; ^5^Department of Neurology, University Hospital Regensburg, Regensburg, Germany

**Keywords:** catheter ablation, stroke, atrial fibrillation, microemboli, transcranial Doppler

## Abstract

**Introduction:**

Left atrial pulmonary vein isolation (PVI) is an accepted treatment option for patients with symptomatic atrial fibrillation (AF). This procedure can be complicated by stroke or silent cerebral embolism. Online measurement of microembolic signals (MESs) by transcranial Doppler (TCD) may be useful for characterizing thromboembolic burden during PVI. In this prospective multicenter trial, we investigated the burden, characteristics, and composition of MES during left atrial catheter ablation using a variety of catheter technologies.

**Materials and methods:**

PVI was performed in a total of 42 patients using the circular-shaped multielectrode pulmonary vein ablation catheter (PVAC) technology in 23, an irrigated radiofrequency (IRF) in 14, and the cryoballoon (CB) technology in 5 patients. TCD was used to detect the total MES burden and sustained thromboembolic showers (TESs) of >30 s. During TES, the site of ablation within the left atrium was registered. MES composition was classified manually into “solid,” “gaseous,” or “equivocal” by off-line expert assessment.

**Results:**

The total MES burden was higher when using IRF compared to CB (2,336 ± 1,654 vs. 593 ± 231; *p* = 0.007) and showed a tendency toward a higher burden when using IRF compared to PVAC (2,336 ± 1,654 vs. 1,685 ± 2,255; *p* = 0.08). TES occurred more often when using PVAC compared to IRF (1.5 ± 2 vs. 0.4 ± 1.3; *p* = 0.04) and most frequently when ablation was performed close to the left superior pulmonary vein (LSPV). Of the MES, 17.004 (23%) were characterized as definitely solid, 13.204 (18%) as clearly gaseous, and 44.366 (59%) as equivocal.

**Discussion:**

We investigated the burden and characteristics of MES during left atrial catheter ablation for AF. All ablation techniques applied in this study generated a relevant number of MES. There was a significant difference in total MES burden using IRF compared to CB and a tendency toward a higher burden using IRF compared to PVAC. The highest TES burden was found in the PVAC group, particularly during ablation close to the LSPV. The composition of thromboembolic particles was balanced. The impact of MES, TES, and composition of thromboembolic particles on neurological outcome needs to be evaluated further. (Clinical Trial Registration: Deutsches Register Klinischer Studien, https://drks-neu.uniklinik-freiburg.de/drks_web/navigate.do?navigationId=trial.HTML&TRIAL_ID=DRKS00003465. DRKS00003465.)

## Introduction

Left atrial catheter ablation leading to electrical pulmonary vein isolation (PVI) is a corner stone therapy for patients with symptomatic atrial fibrillation (AF). PVI targets and eliminates potential electrical triggers of AF located inside the pulmonary veins (PVs). The success rate depends on the type of AF and ranges from 50 to 80% (1). PVI is considered to be a safe procedure when performed by an experienced cardiologist. However, clinically apparent stroke is a fatal complication of this procedure with an incidence of up to 1% (1). More frequently, silent cerebral embolism (SCE) measured by diffusion-weighted MRI (DWI-MRI) has been shown to be a clinically unapparent thromboembolic complication following PVI (2–4). In addition, occurrence of SCE has been linked to different ablation strategies, whereby the pulmonary vein ablation catheter (PVAC) technology seemed to provoke the highest incidence of new DWI lesions (2–6).

Real-time measurement of microembolic signals (MESs) by transcranial Doppler (TCD) is an accepted online biomarker for thromboembolic complications (7–11). So far, microembolus detection using transcranial Doppler (MES-TCD) has been tested in the setting of cardiac disease (i.e., AF), but it has also been used to measure the microembolic burden during invasive procedures (7, 8, 10). Recently, MES monitoring by TCD was also used as a surrogate marker for stroke risk during left atrial catheter abla-tion for AF (5, 6, 12, 13).

However, the data on the quantity and quality of MES during PVI are sparse. In this multicenter trial, we investigated the associated burden, characteristics, and composition of MES during left atrial catheter ablation and compared the different catheter techniques [PVAC, irrigated radiofrequency (IRF), and cryoballoon (CB)].

## Materials and Methods

### Study Design and Subject Characteristics

This observational, non-randomized, prospective trial was performed at three institutions—Department of Internal Medicine II, University Hospital Regensburg, Department of Electrophysiology, Bad Neustadt, and Department of Cardiology and Angiology, Heart Center Bad Krozingen—between July 2011 and November 2012. Ethics approval was obtained from the University of Regensburg (No. 11-101-0134) before the trial commenced, and the study was registered with an international trial registry in Freiburg, Germany (Deutsches Register Klinischer Studien, No. DRKS00003465). Only patients with AF who were scheduled for PVI alone were included in the study. The main exclusion criteria were patient age of <18 and >80 years, chronic neurological disease, diagnosis of dementia, prior left atrial ablation, severe claustrophobia, and implanted cardiac devices (contraindicated for MRI). Informed written consent was obtained from all patients before inclusion in the study.

### Ablation Procedure and Techniques

According to each center’s preference, three different ablation techniques (PVAC, IRF, and CB) were applied to achieve PVI. All techniques were performed in a standardized manner and have been described previously (4). Oral anticoagulation (OAC) therapy was required for at least 4 weeks prior to the procedure to minimize the risk of left atrial thrombus. Ablation was performed under continuous OAC therapy (INR > 2 when using Coumadin). During the study period, a new orally administered direct thrombin inhibitor (dabigatran etexilate, Pradaxa^®^, Boehringer Ingelheim, Ingelheim, Germany) was approved for anticoagulation in patients with non-valvular AF; patients taking this drug were regarded as having full anticoagulation. During the procedure, an activated clotting time (ACT) level >300 s was required. All patients received transesophageal echocardiography to rule out the presence of left atrial thrombus formation.

#### Ablation with the PVAC

Phased RF ablation was performed using the Pulmonary Vein Ablation Catheter^®^ technology (PVAC, Medtronic, Minneapolis, MN, USA). After transseptal access had been obtained, a 12-French steerable sheath was placed. The sheath was flushed throughout the procedure, with maximum flushing occurring during the process of loading and unloading the PVAC. Ablation was started when the ACT was >300 s and was performed using either a 4:1, 2:1, or 1:1 energy mode for 60 s per application with a target temperature of 60°C. PVAC ablation was continued until isolation of each PV was successful; this was confirmed by an entrance block using differential pacing and by evidence of an exit block. It is important to note that the PVAC procedure was not performed according to ERACE criteria (14). Selection of the energy mode, electrode pairs, and the interruption of energy delivery were made according to the initial protocol and each center’s preference. In addition, Genius 14.3 software without PVAC gold technology was employed.

#### Irrigated Radiofrequency (IRF) Ablation

Conventional PVI was performed using an irrigated-tip catheter (NaviStar Thermocool or SmartTouch, Biosense Webster, Dia-mond Bar, CA, USA) and a deflectable, decapolar mapping catheter (Lasso-NAV, Biosense Webster). After transseptal access had been obtained, the geometry of the left atrium was constructed by three-dimensional FAM mapping with the Carto 3 System (CARTO™, Biosense Webster). Point-by-point irrigated radiofrequency ablation was performed to encircle the right and left PVs in pairs. The end-point of the procedure was the electrical isolation of all PVs, which was confirmed by the entrance block and differential pacing. No further ablations were carried out in the left atrium.

#### Ablation with CB

One single transseptal puncture was made, after which a steerable sheath (Flexcath, Medtronic CryoCath LP, Pointe-Claire, QC, Canada) was placed in the left atrium. Ablation was performed using a double-coated over-the-wire CB (Arctic Front, Medtronic). The inner lumen of the CB was connected to a continuous pressure monitoring system. Balloon size was selected in accordance with the diameters of the PVs, as measured by transesophageal echocardiography. In general, a 28-mm balloon was used. The deflated CB was advanced and inflated in front of the venous ostium. After inflation, the balloon was advanced to achieve occlusion of the PV. Occlusion was verified by application of a contrast agent. Each PV was frozen twice over 5 min in the best position for occlusion. During ablation of the right PVs, continuous phrenic nerve pacing was performed from the superior vena cava to promptly detect phrenic nerve injury. If the PV dimensions were too heterogeneous, the size of the balloon was modified to ensure proper occlusion. In case of persistent conduction after CB ablation, electrical isolation was completed segmentally.

### Microembolus Detection Using TD

At all centers, MES-TCD was performed during the ablation procedure using the same equipment (Doppler Box, DWL, Germany). A teaching session was held prior to study initiation. Continuous bilateral insonation in the middle cerebral artery (MCA) territory was attempted with the patient in supine position for cardiac intervention. In accordance with the international consensus recommendations, the sample gate was 8 mm and the Doppler gain was reduced to exhibit only faint Doppler spectra.

**Figure 1:**
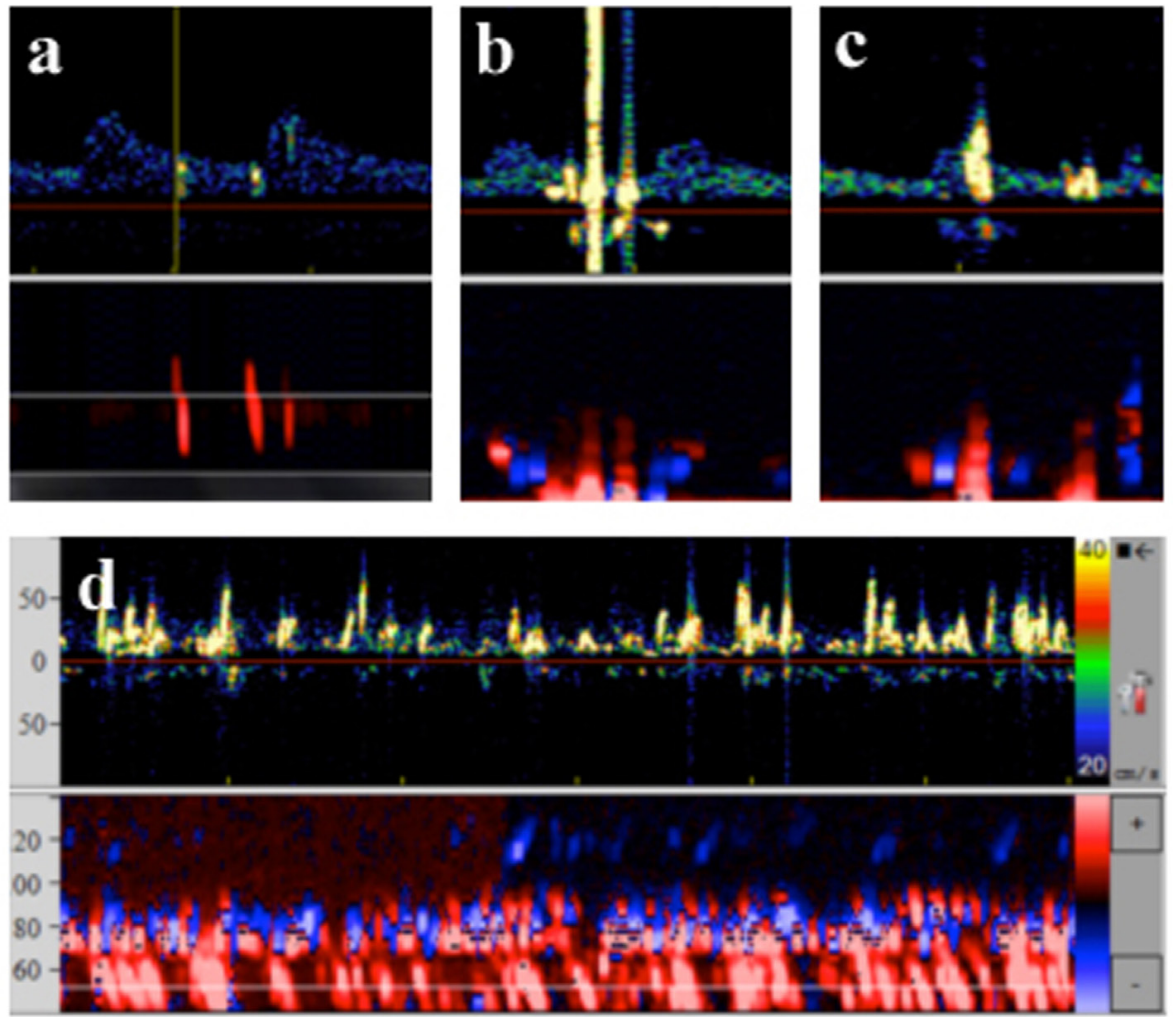
**Transcranial Doppler data and typical characteristics of microembolic signal**. **(A)** Solid emboli: stays within the Doppler spectrum, multigating confirms movement through different depths. **(B)** Gaseous emboli: exceeds the Doppler spectrum, biphasic, multigating reveals signals at one depth only. **(C)** Equivocal emboli: exceeds the Doppler spectrum, but unidirectional, multigating reveals signals at one depth only. **(D)** Thrombembolic shower: signals move at low velocity, continuous embolic activity, oblique multigated signals reveal movement at different depths.

Blinded off-line analysis of the recorded MES-TCD data was performed in a single center by two different neurologists familiar with the technique according to standard criteria (15). In previous studies, application of the implemented gaseous/solid differentiation and detection algorithm was found to be unreliable, probably due to the magnitude and dense number of signals as well as frontend overload (16–18). Thus, manual analysis of MES comprised (i) listening to each signal and (ii) watching each signal on screen at highest speed. Ratings were made without using a decibel threshold. Single countable MES were summed up to a total MES count. Definitely “solid” emboli were strictly unidirectional within the Doppler spectrum and had an acoustic impedance >8 dB over baseline. Clearly “gaseous” emboli were characterized as large and high-intense bidirectional signals exceeding the Doppler spectrum probably due to stimulated acoustic emission from bubble bursts by the incident ultrasound wave (19). When signals did not meet the strict criteria for solid or gaseous emboli they were classified as “equivocal.” In addition, periods with a huge amount of MES lasting longer than 30 s were classified as thromboembolic shower (TES) (Figure 1), as this phenomenon may be associated with a higher risk for cerebral damage. To determine the impact of the ablation site on occurrence of TES, appearance of TES was assigned to the ablated PV [left superior pulmonary vein (LSPV), left inferior pulmonary vein (LIPV), right superior pulmonary vein (RSPV), and right inferior pulmonary vein (RIPV)].

### Core Lab Analysis and Clinical Follow-up

All data were collected by each center and sent to the University Hospital Regensburg for core lab analysis. Off-line analysis of the recorded MES-TCD data was performed as described above by two neurologists. Clinical neurological events were monitored during the patients’ stay in hospital.

### Statistical Analysis

Non-parametric statistical methods were applied in an exploratory manner using SAS^®^, release 9.3 (SAS Institute Inc., Cary, NC, USA) on a Microsoft^®^ Windows^®^ 7 Professional platform. The alpha level for testing was set to 5%, two-sided without correction for multiplicity. Summary statistics were determined. The Wilcoxon rank sum test was carried out for pairwise group comparison of events. In addition, a potential difference in number of TES events with regard to the site of ablation was investigated using the Wilcoxon signed rank test. Furthermore, for the investigation of categorical variables Fisher’s exact test was used. Homogeneity between groups was investigated using the non-parametric Kruskal–Wallis test for continuous variables. This was done taking the ablated structure in each patient into account. The bar charts show the median values or mean values if the group-wise median values are equal to 0.

## Results

### Patient Characteristics

Between July 2011 and November 2012, 42 patients were eligible for TCD analysis. Table 1 shows the patient characteristics and clinical baseline parameters according to the ablation technique used.

**Table 1:** **Table shows baseline and procedural (*italics*) parameters**.

Baseline/*procedural* parameters	Pulmonary vein ablation catheter (PVAC)	IRF	Cryoballoon (CB)	*p* Value
Patients (*n*)	23	14	5	–
Sex male/female	13/10	11/3	4/1	0.41
Mean age	68 ± 8	65 ± 7	63 ± 17	0.53
Atrial fibrillation (AF) type paroxysmal/chronic (*n*)	21/2	8/6	2/3	0.01
Coronary artery disease (*n*)	5	3	0	0.73
Hypertension (*n*)	13	11	3	0.38
Left ventricular hypertrophy (*n*)	7	2	4	0.02
Diabetes (*n*)	4	1	0	0.81
Left ventricular ejection fraction (%)	58 ± 6	56 ± 7	60 ± 5	0.72
Mean left atrial diameter (mm)	44 ± 6	44 ± 12	39 ± 4	0.25
Type of oral anticoagulation (Coumadin/Dabigatran)	16/7	12/2	5/0	0.36
*Ablations performed at University Hospital Regensburg*	*23*	*3*	*0*	
*Ablations performed at Heart Center Bad Krozingen*	*0*	*5*	*0*	*<0.0001*
*Ablations performed at Heart Center Bad Neustadt*	*0*	*6*	*5*	
*Bilateral insonation (yes/no) of middle cerebral artery*	*17/6*	*14/0*	*5/1*	*0.08*
*Total procedure time (min)*	*174* ± *77*	*153* ± *48*	*169* ± *12*	*0.74*
*Fluoroscopy time (min)*	*38* ± *21*	*26* ± *9*	*20* ± *7*	*0.36*
*Energy applications (n)*	*24* ± *10*	*52* ± *38*	*9* ± *2*	*0.001*
*Power (W)*	–	*36* ± *4*	–	–
*Temperature (°C)*	–	*43* ± *4*	–	–

*Homogeneity between groups was investigated using the non-parametric Kruskal–Wallis test for continuous variables and Fisher’s exact test for categorical variables. There was a significant difference regarding AF type, left ventricular hypertrophy, employed ablation technique/center, and number of energy applications*.

### Ablation Procedure

Pulmonary vein isolation was performed using PVAC in 23 patients, IRF in 14 patients, and CB in 5 patients. The mean length of the procedure was 174 ± 77 min in the phased RF (PVAC) group, 153 ± 48 min in the IRF group, and 169 ± 12 min in the CB group; the mean fluoroscopy time was 38 ± 21, 26 ± 9, and 20 ± 7 min, respectively. The mean number of energy deliveries for successful isolation of all PVs was 24 ± 10 using PVAC, 52 ± 38 using IRF, and 9 ± 2 using CB (*p* < 0.001). When using the PVAC, the 2:1 energy setting was employed more frequently than the 4:1 mode (*n* = 363 vs. *n* = 169). Complete isolation could be achieved in all PVs. No additional left atrial ablation was performed. Table 1 shows the procedural parameters for each ablation technique.

### MES Count and Differentiation

**Figure 2:**
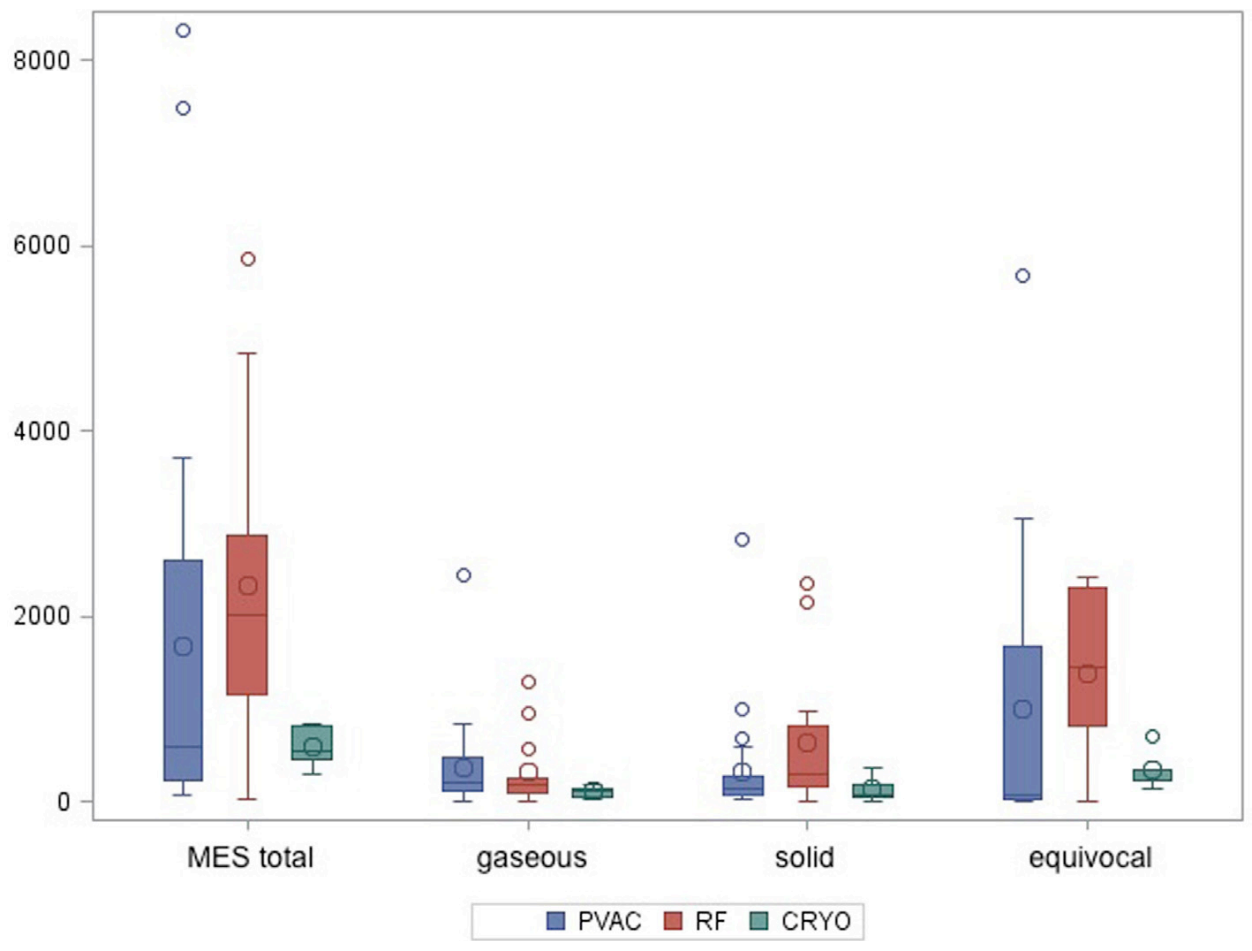
**Boxplot shows median values of total microembolic signal (MES) burden and subdivision of MES according to the different ablation techniques**.

Bilateral insonation of the MCA was feasible in *n* = 35 patients. In *n* = 7 patients, for technical reasons, only unilateral insonation could be achieved. Unilateral insonation was present in six patients treated with PVAC technology at the University Hospital of Regensburg and in one patient treated with CB at the Heart Center Bad Neustadt. A total of 74.574 MES were recorded in 42 patients during the ablation procedures. Of those, 17.004 (23%) MES were characterized as solid, 13.204 (18%) as gaseous, and 44.366 (59%) as equivocal. There was a high variation in the number of MES per patient (lowest with *n* = 21 up to *n* = 8,317). Figure 2 shows the median values of MES and the MES subdivision related to the different ablation techniques. Wilcoxon rank sum test indicates a significantly lower incidence of total and equivocal MES in the CB group compared to the IRF group. Pairwise comparison of the remaining cases did not reach statistical significance. However, there was a tendency toward lower MES burden (total and solid) in the PVAC group compared to the IRF group (Table 2). When using the PVAC, MESs usually occurred 10–15 s after energy delivery had started. We also observed MESs when manipulating/rotating the PVAC after termination of energy delivery.

**Table 2:** **Total microembolic signal (MES) burden, MES differentiation, and thromboembolic shower (TES) burden are represented as median and mean values**.

	Pulmonary vein ablation catheter (PVAC)	IRF	Cryoballoon (CB)	PVAC vs. IRF	PVAC vs. CB	IRF vs. CB
Pts. (*n*)	23	14	5	–	–	–
MES total median/mean ± SD	602/1,685 ± 2,255	2,009/2,336 ± 1,654	545/593 ± 231	*p* = 0.08	*p* = 0.81	*p* = *0.007*
MES solid median/mean ± SD	147/330 ± 593	293/628 ± 770	82/135 ± 137	*p* = 0.08	*p* = 0.45	*p* = 0.13
MES gaseous median/mean ± SD	208/360 ± 511	187/327 ± 386	119/106 ± 67	*p* = 0.84	*p* = 0.13	*p* = 0.20
MES equivocal median/mean ± SD	74/994 ± 1,448	1,439/1,380 ± 799	345/351 ± 213	*p* = 0.12	*p* = 0.71	*p = 0.01*
Pts. with TES (*n*)	10	2	0	–	–	–
TES median/mean ± SD	0/1.5 ± 2	0/0.4 ± 1.3	0/0	*p* = *0.04*	*p* = 0.08	*p* = 0.61

*P values are based on the non-parametric Wilcoxon rank sum test for pairwise comparison between the ablation groups. P values are calculated by pairwise comparison between the ablation groups*.

*Pts., patients*.

### TES and Assignment to Ablation Site

**Figure 3:**
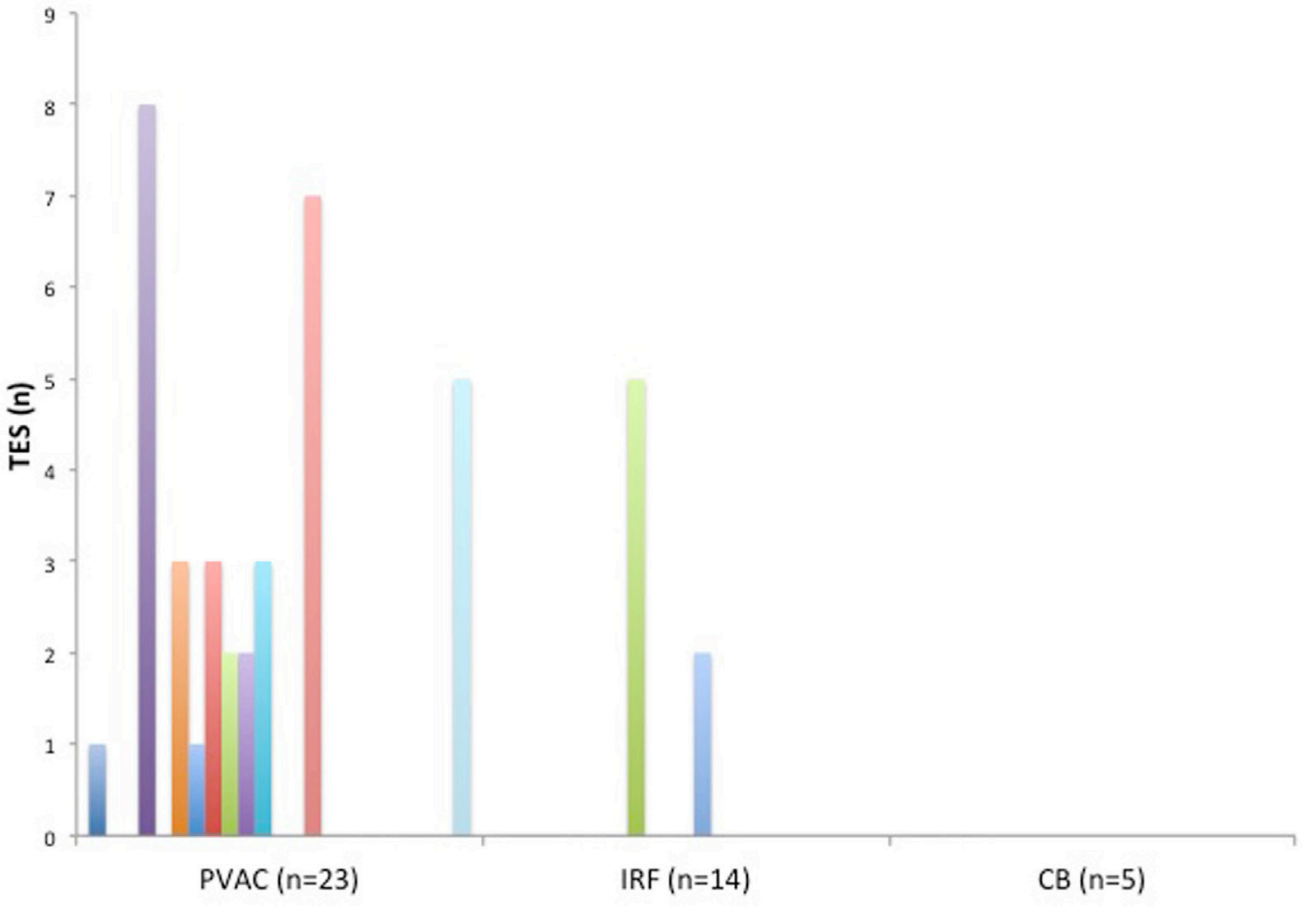
**The burden (*n*) of thromboembolic shower (TES) in each patient related to the ablation technique**. TES were found in 10 patients of the pulmonary vein ablation catheter (PVAC) group and in 2 patients of the IRF group. No TES were seen in the cryoballoon (CB) group.

**Figure 4:**
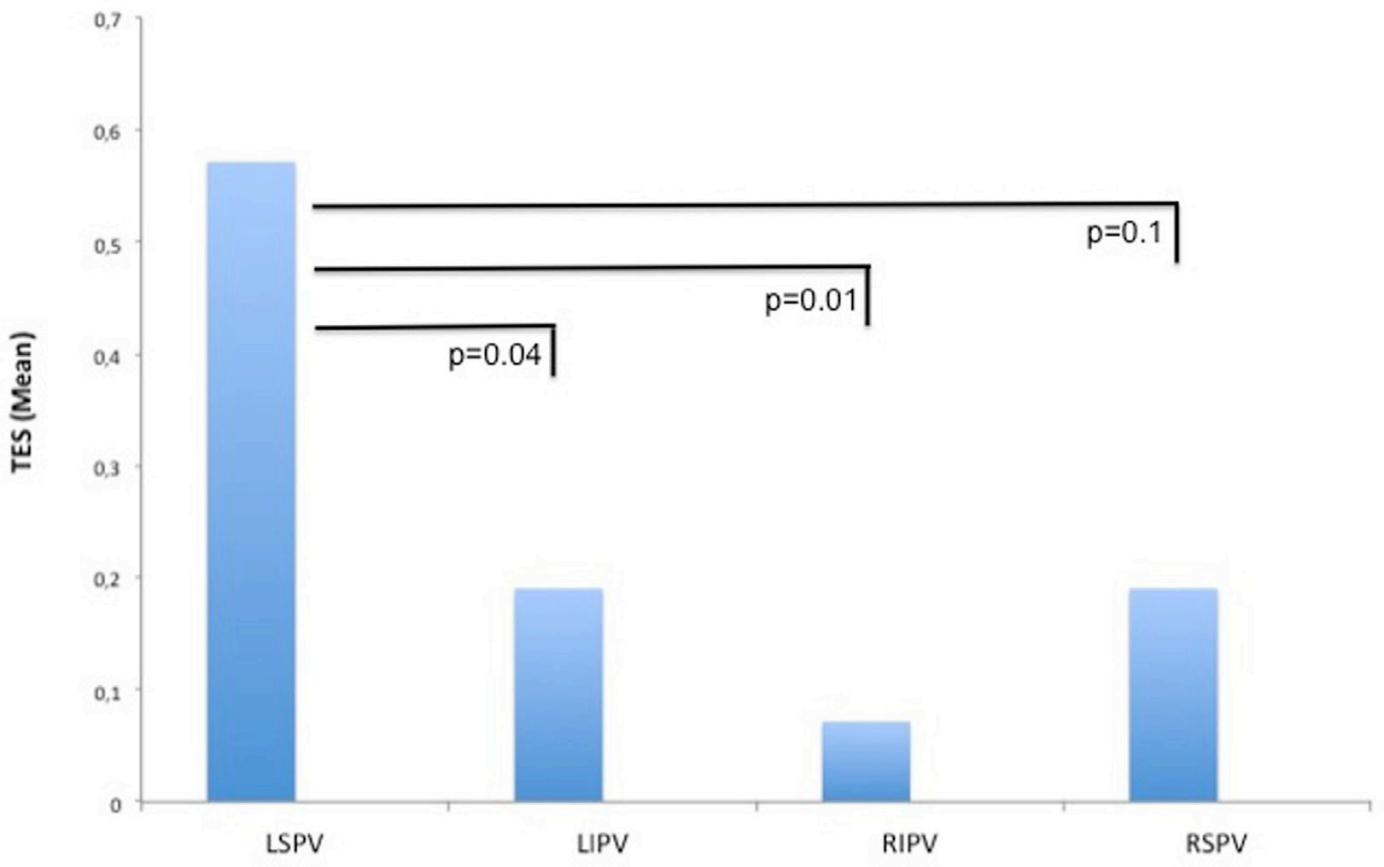
**Thromboembolic shower (TES) burden related to the ablation site**. When ablating the left superior pulmonary vein (LSPV), the burden of TES at this site was significantly higher compared to the left inferior pulmonary vein (LIPV) and right inferior pulmonary vein (RIPV).

A total of 42 TESs were registered in 12 different patients during the ablation procedures. The majority of the TESs (*n* = 35 in 10 patients) were recorded in the PVAC group. Only seven TESs were found in two patients in the IRF group and no TESs were registered in the CB group (Figure 3). Pairwise comparison showed a significantly higher TES burden in the PVAC group compared to the IRF group. There was a tendency toward a lower TES burden in the CB group compared to the PVAC group, which was not statistically significant (Table 2). Observing the occurrence of TESs pertaining to the ablation site, we found a significantly higher rate of TES when ablating the LSPV compared to the left infe-rior pulmonary vein and right inferior pulmonary vein (Figure 4).

### Clinical Outcome

None of the patients suffered a clinical stroke during their hospital stay; this also applied to the patient with the highest (*n* = 8,317) MES burden. However, one patient in the PVAC group reported a subjective visual disturbance lasting several seconds, which was assessed as a migraine with aura. This patient had a low total MES burden (*n* = 174) and one TES.

## Discussion

Evidence of SCE illustrated by DWI-MRI has drawn attention to an important feature of clinically asymptomatic neurological complications following PVI. The use of the PVAC, in particular, seemed to provoke a high incidence of new DWI lesions (2, 3). However, cerebral MRI only documents thromboembolic events after and not during the ablation procedure. Online measurement of MESs by TCD is a useful tool for learning more about the thromboembolic burden during left atrial catheter ablation. So far, there are little data available on this subject.

This multicenter study investigated the burden and characteristics of MES during PVI using three different ablation techniques. The results show that: (i) all ablation techniques applied in this study generated a relevant number of MESs. The total MES burden was significantly higher in the IRF group compared to the CB group and showed a tendency toward a higher burden in the IRF group compared to the PVAC group. (ii) TESs occurred more often in the PVAC group compared to the IRF group. In the small number of patients treated with CB, no TESs were observed. TESs were seen most frequently when ablation was performed close to the LSPV. (iii) This study provides the first data on subdivision of MES (gaseous vs. solid vs. equivocal) by manual and not automated analysis, showing a balanced distribution of solid and gaseous MES burden.

Our study adds to the findings on total thromboembolic burden and is in line with previous studies demonstrating a lower total MES count in the CB group and a higher MES burden when using IRF or the PVAC with an initial protocol (6, 13, 20, 21). MESs were observed during energy delivery as well as during catheter manipulation immediately after ablation. Remarkably, our study even shows a higher MES burden by trend in the IRF group compared to the PVAC group. This is in contrast to previous studies, which demonstrated a median MES ranging from 646 ± 449 to 1,404 ± 98 in the IRF group (13, 20). By comparison, the median MES burden in the IRF group in our study (2,336 ± 1,654) was high. This may be attributed to the higher number of energy applications, the maximum power, and maximum energy settings used in our study. These parameters seem to correlate with MES burden and upper values may be responsible for a higher MES count (13). In addition, Kochhäuser et al. (20) found more MES during catheter replacement in the IRF group compared to the PVAC group, which might have been a relevant factor in the present study. However, our finding strongly emphasizes the need to reduce MES when using the IRF or PVAC technology.

Although MES burden was higher in the IRF group we found more TES when using the PVAC technology compared to IRF. In the small number of patients in the CB group, we found no TES. As TES potentially may cause cerebral damage to a greater extent, this finding may correspond to the higher incidence of SCE measured by MRI following PVAC ablation (2, 3) and supports the attempt to reduce embolic signals by deactivation of the distal electrodes, optimizing catheter-tissue contact, and employment of the newest Genius generator software (6, 22). Interestingly, TES occurred mainly when ablating at the region of the LSPV. This is in line with the study conducted by Nagy-Balo et al. (23) who found more MES during ablation close to the left-sided PVs. Proximity of the LAA creating a complex anatomy between LSPV and the ridge and/or a steep catheter position based on the direction of the inferior caval vein and transseptal access may lead to alternating contact force and/or to an overlap of electrodes 1 and 10 during PVAC procedures. These conditions are known mechanisms of microembolization and may increase the incidence of TES.

The composition of the thromboembolic burden may be a relevant factor pertaining to cerebral damage, as gaseous MES might be less harmful than solid particles. Thus, subdivision of MES into gaseous and solid may be of diagnostic relevance. Only a small number of studies identify gaseous particles as the main source of microembolization during left atrial catheter ablation (6, 21). However, the subdivision was done using an automated algorithm, which has not yet been sufficiently evaluated for precision (16–18). Performing a manual analysis, we found a homogeneous distribution of solid and gaseous MESs, in which IRF shows a tendency to elicit more solid particles compared to PVAC. Solid particles may occur due to charring and a more aggressive ACT target during ablation could reduce the solid component. However, current studies evaluating this issue did not reach statistical significance and thus the anticoagulation protocol in TCD studies needs to be evaluated further (21).

Importantly, the impact of documented MES during PVI on neurological outcome is still unclear. Kilicaslan et al. report an acute neurological complication rate of 36% in patients with MES >3,000 (12). Another study found a correlation of MES with a subtle neuropsychological deficit post ablation (20). None of the patients in our study, even those with MES >3,000 (*n* = 7), suffered a clinical stroke during their stay in hospital. One patient with migraine episode showed only a low MES burden. Thus, the potential context of MES and neurological impairment should be investigated more extensively.

As clinically unapparent thromboembolic events may be a potential complication of PVI, the indication for left atrial catheter ablation has to be considered properly. However, even in the light of thromboembolic risk, PVI remains an important treatment option for patients with symptomatic AF, as AF burden can severely impair the quality of life. There is need for further studies elaborating the risk of stroke during the ablation procedure.

### Limitations

This study has several limitations: (1) we performed a non-randomized study with a small sample size in the CB group which hampers the possibility to draw firm conclusions concerning the employed CB technology. CB ablation was an upcoming technology during the study period and was not routinely performed at all study centers. Hence, the study protocol did not pretend the catheter technology to allow for the best expertise at each study center. This generated a small number in the CB cohort, limiting statistical testing pertaining to this group. It is also unclear whether TES would have occurred if the CB patient cohort was larger. Focusing on MES alone, our CB data are in line with other studies and should not be withheld. (2) According to the non-randomized character of this study, variables as AF type (PAF/CAF), left ventricular hypertrophy, employed ablation technique/center, and number of energy applications were not equally distributed in the different groups, which creates heterogeneity and may influence the burden of MES/TES during the procedure. (3) MES in patients with unilateral insonation was not counted differently to not witheld this data. However, this may be a source of bias, as MES of the contralateral side is not incorporated in these patients. (4) MES is registered during the entire ablation procedure without discriminating energy delivery from catheter manipu-lation or transseptal puncture. Thus, no assignment of MES to single steps of the procedure can be made. However, “time stamping” is available for TES recording. (5) The standard evaluation of MES is off-line assessment by an expert (17), but the human factor of manual analysis in our study needs to be considered.

## Conclusion

The present study demonstrates a significant difference in total MES burden using IRF compared to CB and a tendency toward a higher burden using IRF compared to PVAC. This highlights the need to reduce MES also when using devices other than the PVAC, i.e., IRF. The most intense TESs, indicating a severe and sustained thromboembolic burden, were found in the PVAC group, particularly during ablation close to the LSPV. The thromboembolic composition (solid vs. gaseous) was analyzed manually for the first time and was balanced. The impact of MESs and TESs on neurological outcome needs to be evaluated further.

## Ethics Statement

This study was carried out in accordance with the recommendations of the ethics committee (Prof. H. Helbig, PD Dr. K. Ittner, W. Stelzl, Prof. R. Witzgall) of the University of Regensburg, with written informed consent from all subjects. All subjects gave written informed consent in accordance with the Declaration of Helsinki. The protocol was approved by the ethics committee of the University of Regensburg.

## Author Contributions

CB: conception or design of the work, data collection, data analysis and interpretation, drafting the article, and final approval. TD and TA: data collection, data analysis and interpretation, criti-cal review, and final approval. AS, HL, and SF: data collection, critical review, and final approval. DB: data analysis and interpretation, critical review, and final approval. LM: critical review and final approval. FS: data collection, data analysis and interpretation, drafting the article, and final approval.

## Conflict of Interest Statement

There are no ethical problems and no conflicts of interest for any of the authors related to the material. The research was conducted in the absence of any commercial or financial relationship that could be construed as a potential conflict of interest. Regarding this observational study an infor-med written consent was obtained from all patients before inclusion in the study.

## Abbreviations

ACT, activated clotting time; AF, atrial fibrillation; CB, cryoballoon; IRF, irrigated radiofrequency; LIPV, left inferior pulmonary vein; LSPV, left superior pulmonary vein; MESs, microembolic signals; PVAC, pulmonary vein ablation catheter; PVI, pulmonary vein isolation; PV, pulmonary vein; RIPV, right inferior pulmonary vein; RSPV, right superior pulmonary vein; SCE, silent cerebral embolism; TCD, transcranial Doppler; TESs, thromboembolic showers.
